# An optimized immunohistochemistry protocol for detecting the guidance cue Netrin-1 in neural tissue

**DOI:** 10.1016/j.mex.2017.12.001

**Published:** 2017-12-13

**Authors:** Samer Salameh, Dominique Nouel, Cecilia Flores, Daniel Hoops

**Affiliations:** Department of Psychiatry, Douglas Mental Health University Institute, McGill University, 6875 LaSalle Boulevard, Montreal, Quebec, H4H 1R3, Canada

**Keywords:** AF, Alexa Fluor, BSA, bovine serum albumin, DAPI, 4′, 6-diamidino-2-phenylindole, HRP, horseradish peroxidase, IF, immunofluorescence, IHC, immunohistochemistry, NDS, normal donkey serum, PB, phosphate buffer, PBS, phosphate-buffered saline, RT, room temperature, SDS, sodium dodecyl sulfate, SNR, signal-to-noise ratio, TB, tris buffer, TBS, tris-buffered saline, TH, tyrosine Hydroxylase, Sodium dodecyl sulfate, Citrate buffer, Phosphate buffer, Immunofluorescence, Antigen retrieval, Signal intensity, Signal-to-noise ratio, Brain, Neuroscience

## Abstract

Netrin-1, an axon guidance protein, is difficult to detect using immunohistochemistry. We performed a multi-step, blinded, and controlled protocol optimization procedure to establish an efficient and effective fluorescent immunohistochemistry protocol for characterizing Netrin-1 expression. Coronal mouse brain sections were used to test numerous antigen retrieval methods and combinations thereof in order to optimize the stain quality of a commercially available Netrin-1 antibody. Stain quality was evaluated by experienced neuroanatomists for two criteria: signal intensity and signal-to-noise ratio. After five rounds of testing protocol variants, we established a modified immunohistochemistry protocol that produced a Netrin-1 signal with good signal intensity and a high signal-to-noise ratio. The key protocol modifications are as follows:

• Use phosphate buffer (PB) as the blocking solution solvent.
• Use 1% sodium dodecyl sulfate (SDS) treatment for antigen retrieval.

The original protocol was optimized for use with the Netrin-1 antibody produced by Novus Biologicals. However, we subsequently further modified the protocol to work with the antibody produced by Abcam. The Abcam protocol uses PBS as the blocking solution solvent and adds a citrate buffer antigen retrieval step.

## Graphical abstract

**Figure:**
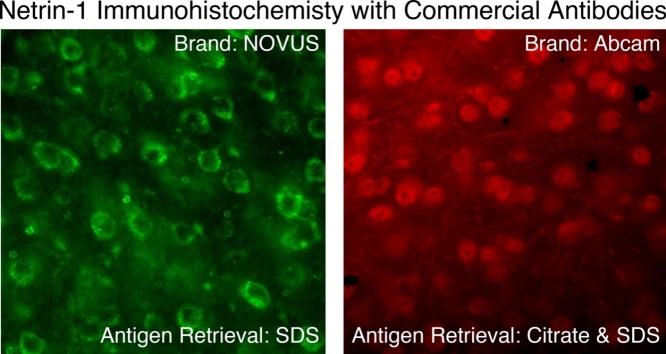

## Specifications Table

**Table:** 

Subject area	Neuroscience
More specific subject area	*Protein Detection*
Method name	*Immunohistochemistry Protocol for Detecting Netrin-1*
Name and reference of original method	*Method: Immunohistochemistry*
	*Reference:* Cooper, P. (1997) [1]. *Immunohistochemistry. In Neuroscience* Methods: *A Guide for Advanced Students (pp. 131–136). Amsterdam, The Netherlands: Harwood Academic Publishers.*
Resource availability	*Chicken anti-netrin-1 primary antibody (catalog #NB100-1605, Novus Biologicals, RRID: AB_2298755)*
	*Rabbit anti-netrin-1 primary antibody (catalog #ab126729, Abcam, RRID: AB_11131145)*

## Method details

### Netrin-1 fluorescence immunohistochemistry protocol (Novus brand antibody)

1. Perfuse mice using heparinized saline followed by 4% paraformaldehyde.
2. Incubate brains overnight in 4% paraformaldehyde at 4 °C.
3. Section brains at 35 μm using a vibratome and collect brain sections in phosphate-buffered saline (PBS).
4. Place sections in a 1% sodium dodecyl sulfate (SDS) solution for 5 min on an orbital shaker at room temperature (RT).
5. Rinse sections in phosphate buffer (PB) 3 times, 5 min each, on an orbital shaker at RT.
6. Incubate sections in blocking solution (2% bovine serum albumin and 0.2% tween in PB) for 1 h on an orbital shaker at RT.
7. Dilute chicken anti-netrin-1 primary antibody (catalog #NB100-1605, Novus Biologicals) 1:500 in blocking solution.
8. Incubate sections in primary antibody solution for 4 nights/3 days on an orbital shaker at 4 °C.
9. Rinse sections in PB 3 times, 5 min each, on an orbital shaker at RT.
10. Dilute goat anti-chicken Alexa Fluor 488 secondary antibody (catalog #A-11039, NOVUS Biologicals) 1:500 in blocking solution.
11. Incubate sections in secondary antibody solution for 1 h on an orbital shaker at RT.
12. Rinse sections in PB 3 times, 5 min each, on an orbital shaker at RT.
13. Mount sections on gel-coated slides and cover-slip with mounting medium (Vectashield Hard-Set Mounting Medium with DAPI; catalog #H-1500, Vector Laboratories).

We store the Novus brand Netrin-1 antibody at −20 °C, diluted 50% in glycerol.

### Abcam brand netrin-1 antibody protocol modifications

The following modifications to the protocol described above were necessary to obtain high-quality staining using the antibody produced by Abcam:

1. Use PBS in place of PB
2. Place the sections in citrate buffer in a water bath (100 °C) for 5 min, and then rinse them in PBS 3 times, 5 min each, on an orbital shaker at RT prior to the 1% SDS treatment.

We store the Abcam brand Netrin-1 antibody undiluted at −20 °C in single-use aliquots.

## Method validation

### Rationale

Attempts to characterize Netrin-1 expression using fluorescence immunohistochemistry (IHC) have been largely unsuccessful. Recently, we undertook a multi-step blinded IHC optimizing procedure resulting in a simple protocol for high quality IHC Netrin-1 staining. We tested several antigen retrieval methods, reagent variations, and combinations thereof using a careful process of elimination involving five rounds of testing.

### Standard IHC protocol

**Fig 1:**
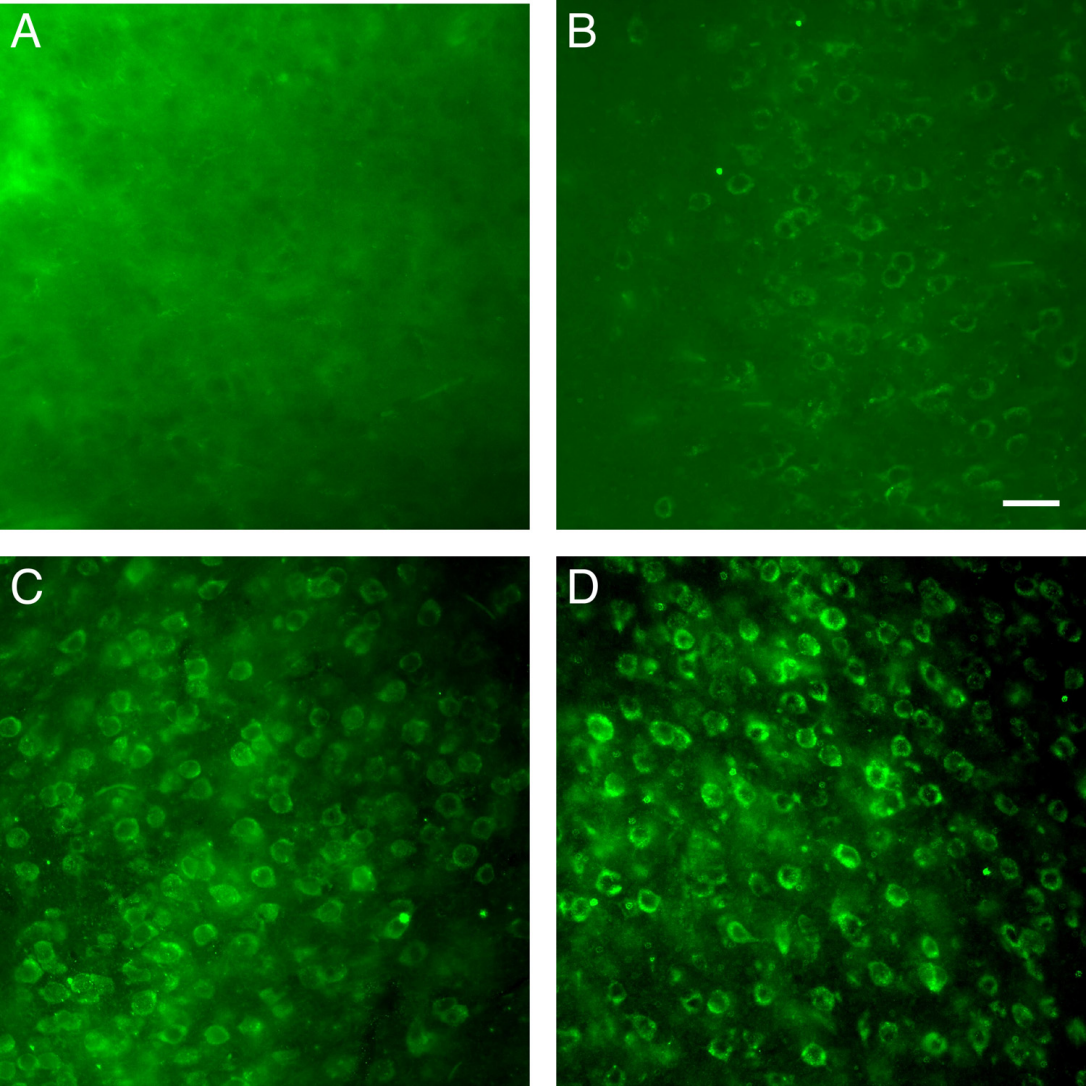
Netrin-1 labelling in the mouse forebrain, with an antibody commercially available from Novus Biologicals, using four different immunohistochemistry (IHC) protocols. ***A***, Netrin-1 signal is not discernable from background noise when using our group’s standard IHC protocol. ***B***, Modifying our group’s IHC protocol by adding a heat treatment results in weak detection of Netrin-1. ***C***, Combining the heat treatment with an SDS treatment enhances the Netrin-1 signal. ***D***, SDS treatment without the heat treatment reduces the background noise and does not appear to impact the intensity of the Netrin-1 signal. ***A–D***, Netrin-1 IF was viewed under green fluorescence at ×40 magnification. Scale bar: 20 μm. (For interpretation of the references to colour in this figure legend, the reader is referred to the web version of this article.)

First, we established whether Netrin-1 could be detected using our group’s standard IHC protocol. This protocol consists of steps 6–13 as described above except that the blocking solution is made with PBS, not PB, and all rinses are also performed using PBS. This protocol does not result in Netrin-1 staining (Fig. 1a).

### Protocol modifications

We proceeded to systematically alter the standard protocol, first to determine whether we could detect Netrin-1, and subsequently to optimize the signal intensity and signal-to-noise ratio (SNR). We compiled a list of possible method alterations by consulting experts in IHC and published peer-reviewed articles that focus on antigen retrieval [[2], [3], [4], [5]]. These alterations were:

1. Use phosphate buffer (PB), phosphate-buffered saline (PBS), tris buffer (TB), or tris-buffered saline (TBS) as the blocking solution solvent.
2. Heat the sections for 5 min at 90 °C or 100 °C in either blocking solution solvent (PBS, PB, TBS, or TB) or a citrate buffer prior to blocking.
3. Increase the concentration of tween in the blocking solution.
4. Perform the primary antibody incubation at room temperature instead of 4 °C.
5. Use 3% or 5% milk as a blocking agent in place of bovine serum albumin.
6. Use 5% milk as a blocking agent during the blocking incubation and then 3% milk as a blocking agent during the primary antibody incubation.
7. Add 2% normal donkey serum (NDS) to the blocking solution.
8. Place the sections in 1% SDS for 5 min prior to blocking incubation.
9. Use horseradish peroxidase (HRP) conjugated anti-chicken secondary antibody (catalog #303-035-003, Jackson ImmunoResearch Laboratories), and then incubate sections in H_2_O_2_ solution containing TSA Tyramide Reagent (Product #NEL704A001, PerkinElmer).
10. After secondary antibody incubation, incubate the sections in H_2_O_2_ solution for 10 min.

All tests were performed on sections of adult mouse brain tissue, and each protocol variant was tested on at least 30 sections from two mice.

### Protocol optimization results

The Netrin-1 signal of each variant was qualitatively evaluated for signal intensity and SNR by two neuroanatomists with extensive experience in IHC. However, not all possible combinations of the ten aforementioned alterations were tested. We performed five rounds of testing, and the protocol that resulted in the highest quality staining in each round was used as the basis for the next round of tests. We found that the optimal Netrin-1 IHC protocol consists of two crucial alterations to our group’s standard IHC protocol. First, PB is used instead of PBS. Second, the sections are placed in 1% SDS solution, an antigen retrieval step, for 5 min prior to the block incubation. We also found that heating sections prior to blocking resulted in Netrin-1 staining; however, the Netrin-1 signal was weaker than with SDS.

The heat treatments (sub-boiling and boiling) were the first methods we tested that showed promise in detecting Netrin-1 using IHC. The treatments were performed by transferring brain sections into Eppendorf tubes and heating them in a 90 °C (sub-boiling) or 100 °C (boiling) water bath for 5 min. Heating was recommended by IHC experts and peer-reviewed articles as an efficient antigen retrieval method that alters the structure of antigens, making them more detectable by antibodies [4]. Since the pH of the blocking solution solvent has been thought to affect the efficiency of antigen retrieval with heat, we compared the efficiency of heating in PB, a weak base, against citrate buffer, a weak acid [4]. However, we did not observe any difference in staining quality between PB and citrate buffer. Though heating the sections was successful in detecting Netrin-1, the staining was weak and there was low SNR (Fig. 1b). Consequently, we continued to test antigen retrieval methods.

Although SDS treatment was highly recommended as an efficient antigen retrieval method, previous studies only discuss its use as a complement for heat treatment [5]. Moreover, when heat was established as a necessary treatment for Netrin-1 labelling after the second round of protocol testing, SDS was only predicted to enhance its efficiency. Unexpectedly, we found that 1% SDS solution was not only successful in obtaining a Netrin-1 signal in the absence of heat, but it also yielded a more favorable SNR than the heat treatment alone or in combination with SDS (Fig. 1c, d). The SDS treatment was also less time consuming than heat, since it did not require transferring sections into Eppendorf tubes. A drawback of the SDS treatment was that it caused the sections to become more transparent and delicate. Nonetheless, the SDS treatment was the most effective and efficient antigen retrieval method that we tested.

The remaining methods we tested did not impact the quality of Netrin-1 IHC. In the absence of heat or SDS, these methods all failed to show any Netrin-1 signal. In the presence of heat or SDS, they either did not impact the signal quality or reduced it.

We also note that several steps specified by our protocol could potentially be modified without impacting the quality of the staining, and are simply the procedure of choice for our group. These include the secondary antibody used and the method of sectioning. We provide these details in order to maximize reproducibility. Furthermore, a complete and detailed description of our five-round protocol optimization procedure can be found in the Supplementary materials.

### Abcam brand-specific comments

**Fig. 2:**
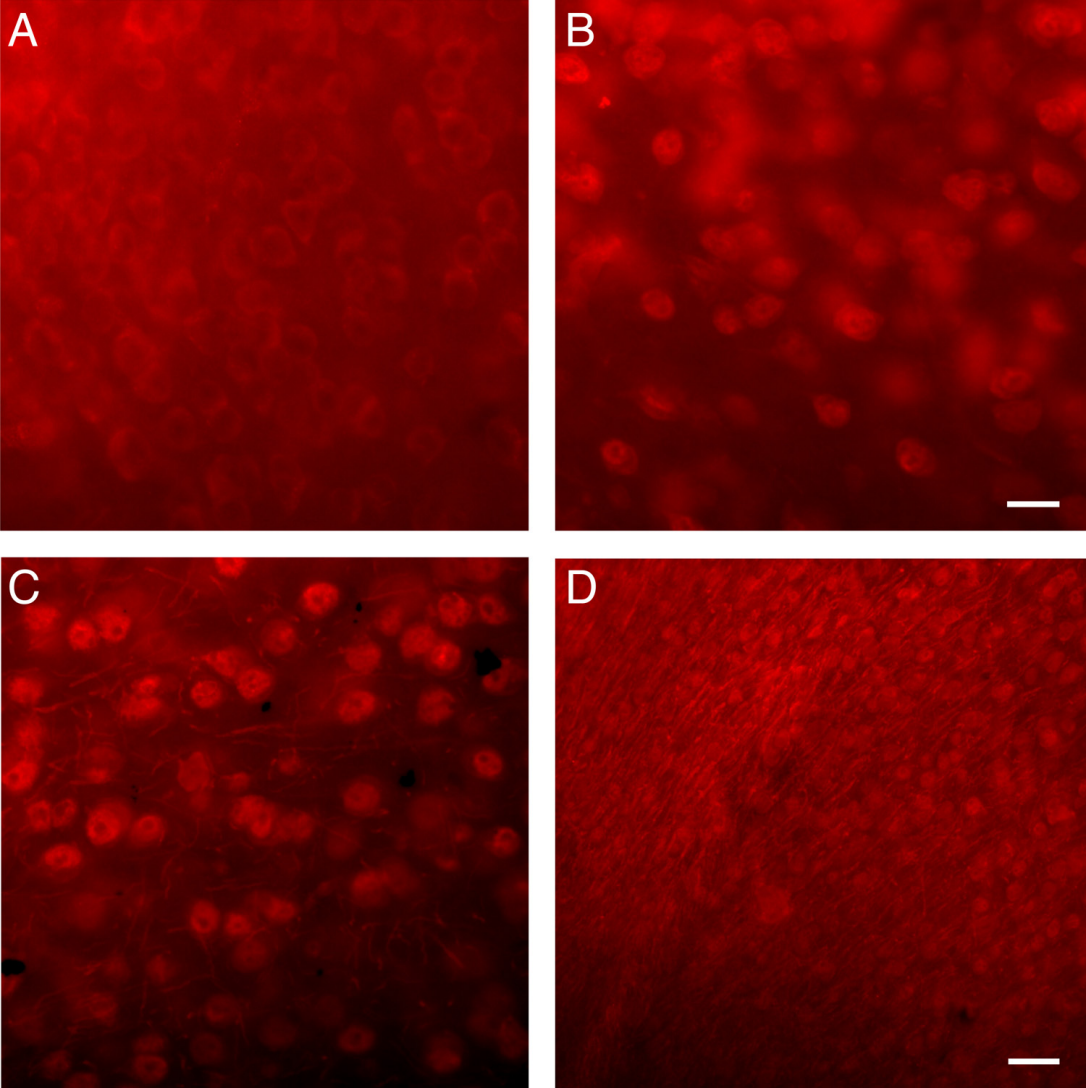
Netrin-1 labelling in the mouse forebrain, with an antibody commercially available from Abcam, using three different immunohistochemistry (IHC) protocols. ***A***, The protocol we developed for use with the antibody produced by Novus Biologicals does not work well with this antibody. ***B***, Netrin-1 signal is enhanced when modifying the protocol by using PBS instead of PB and a citrate buffer heat treatment instead of SDS. ***C***, Adding an SDS incubation step to the protocol used in B enhances the signal further and decreases the background noise. Only when using this protocol were fibers expressing Netrin-1 visible in addition to cell bodies. ***D***, The protocol as used for part **C** allows for visualization of fibers expressing Netrin-1 radiating from the corpus callosum into the cortex. ***A–C***, Netrin-1 IF was viewed under red fluorescence at x 40 magnification. Scale bar: 20 μm. ***D***, Netrin-1 IF was viewed under red fluorescence at ×20 magnification. Scale bar: 40 μm. (For interpretation of the references to colour in this figure legend, the reader is referred to the web version of this article.)

After establishing a successful Netrin-1 IHC protocol using the Netrin-1 antibody produced by Novus Biologicals, we investigated whether our protocol could also accommodate the Abcam brand Netrin-1 antibody (catalog #ab126729). We conducted a single round of protocol modifications to test the Abcam antibody and ultimately found that a heat treatment and the use of PBS were required for optimal staining with this antibody (Fig. 2).

When using this antibody, we noted that the binding affinity was severely diminished by even a single freeze-thaw cycle, and that diluting the antibody in glycerol also appeared to reduce the quality of the stain. Consequently, we recommend using Abcam antibody aliquots that have been frozen continuously at −20 °C, or to keep the antibody at 4 °C, to ensure optimal labelling.

## Animals

All experiments were performed in accordance with the guidelines of the Canadian Council of Animal Care, and all animal procedures were approved by the McGill University/Douglas Hospital Animal Care Committee (Ethics Protocol #7524). Mice were weaned at post-natal day 21 and then housed with same-sex littermates, maintained on a 12-h light-dark cycle (lights on at 0700 h), and given ad libitum access to food and water.

## Additional information

Netrin-1, a 65-kDa protein, plays an important role in the development and maintenance of the central nervous system throughout life [[6], [7], [8], [9]]. Its most well-known function is guiding growing axons to their innervation targets and therefore it is referred to as a “guidance cue protein” [[10], [11], [12]]. However, Netrin-1 also plays a critical role in the wiring events that follow axonal pathfinding, including target recognition, axon arborisation, and synapse formation [[10], [11], [12]]. Despite being a secreted protein located in the extracellular space, recent evidence suggests that Netrin-1 does not act by diffusing into a gradient from a source of secretory cells, but rather by binding to the extracellular matrix and influencing the local decision making processes of growing axons [[7], [8], [13], [14]]. Because of this, determining the localization of Netrin-1 in the brain can provide important information regarding the mechanisms of neural development.
