# Supplementary material for: An optimized immunohistochemistry protocol for detecting the guidance cue Netrin-1 in neural tissue
Source: MethodsX. 2017 Dec 13;5:1–7. doi: 10.1016/j.mex.2017.12.001 (PMC5737949; doi:10.1016/j.mex.2017.12.001)
Supplement: Supplementary file 1 [file mmc1.docx]

**Supplementary Materials 1, Protocol Optimization Procedure**

In order to optimize the IHC protocol for Netrin-1 detection, we tested a number of antigen retrieval methods and combinations thereof using a careful process of elimination involving five rounds of testing.

In the first round of testing protocol variations, we examined two heat treatments: sub-boiling (90°C) and boiling (100°C). Each heat treatment was tested in either phosphate-buffered saline (PBS) or citrate buffer. The other treatments introduced in round 1 were doubling the concentration of tween in the blocking solution and conducting the primary incubation at room temperature (RT; Table 1).

**Table 1: Round 1 Protocol Variants**

| **Variants**  **Steps** | **1** | **2** | **3** | **4** | **5** | **6** |
| --- | --- | --- | --- | --- | --- | --- |
| Collect | Collect brain sections in PBS | | | | | |
| Heat Treatment | No Heat | | Heat the sections for 5 min at **90°C** | | Boiling Treatment: Heat the sections for 5 min at ~**100°C** | |
|  |  |  | In **PBS** | In **citrate buffer** | In **PBS** | In **citrate buffer** |
| Blocking Solution Recipe | 2% BSA and 0.2% tween in PBS | 2% BSA and **0.4% tween** in PBS | 2% BSA and 0.2% tween in PBS | | | |
| Blocking Incubation | 1 hour incubation in block on the orbital shaker at RT | | | | | |
| Primary Antibody Solution | Dilute chicken anti-Netrin-1 antibody at 1:500 concentration and rabbit anti-TH antibody at 1:1000 concentration in block | | | | | |
| Primary Antibody Incubation | Incubate sections in primary antibody solution for 4 nights on the orbital shaker **at RT** | Incubate sections in primary antibody solution for 4 nights on the orbital shaker at 4°C | | | | |
| Wash | Wash sections in PBS 3 times, 5 min each, on the orbital shaker at RT | | | | | |
| Secondary Antibody Solution | Dilute goat anti-chicken (AF488) and donkey anti-rabbit (AF594) secondary antibodies at 1:500 concentrations in block | | | | | |
| Secondary Antibody Incubation | Incubate sections in secondary antibody solution for 1 hour on the orbital shaker at RT | | | | | |
| Wash | Wash sections in PBS 3 times, 5 min each, on the orbital shaker at RT | | | | | |
| Mount and Coverslip | Mount sections on gel-coated slides and cover-slip with DAPI | | | | | |

**Abbreviations:**

PBS: Phosphate buffered saline;

BSA: Bovine Serum Albumin;

RT: Room temperature;

TH: Tyrosine Hydroxylase;

AF: Alexa Fluor;

DAPI: 4’, 6-diamidino-2-phenylindole

No Netrin-1 signal was obtained without heat or boiling, indicating that the double tween and RT primary antibody incubation treatments were ineffective antigen retrieval methods. Furthermore, the signal obtained by heat and boiling was weak and had a low signal-to-noise ratio (SNR). There was no difference between sub-boiling and boiling treatments, nor was there any difference between heating in blocking solution solvent or citrate buffer.

In the second round of testing protocol variations, the standard immunohistochemistry (IHC) protocol was performed with and without heat treatment, the only additional variant being the use of phosphate buffer (PB), tris-buffered saline (TBS), or tris buffer (TB) as a blocking solution solvent. A total of six variants were tested: each solvent with and without heat treatment. We found that PB achieved the optimal level of Netrin-1 immunofluorescence (IF) signal and would be the blocking solution solvent of choice moving forward. Furthermore, the inclusion of a heat treatment in the protocol was necessary to obtain Netrin-1 IF signal.

In the third round of testing protocol variations (Table 2, Figure 1), more treatments were introduced in order to determine which additions would enhance the Netrin-1 signal obtained with heat and PB. These variations were: 1% sodium dodecyl sulfate (SDS), 5% milk, 3% milk, 3% milk for primary incubation and 5% milk for other steps, 2% normal donkey serum (NDS), and horseradish peroxidase (HRP)-conjugated anti-chicken secondary antibody followed by an incubation in H_2_O_2_ solution containing TSA Tyramide Reagent at a concentration of 1:200.

**Table 2: Round 3 Protocol Variants**

| **Variants**  **Steps** | **1** | **2** | **3** | **4** | **5** | **6** | **7** | **8** | **9** | **10** | **11** | **12** | **13** | **14** |
| --- | --- | --- | --- | --- | --- | --- | --- | --- | --- | --- | --- | --- | --- | --- |
| Collect | Collect brain sections in PBS | | | | | | | | | | | | | |
| Wash | Wash sections in PB 3 times, 5 min each, on the orbital shaker at RT | | | | | | | | | | | | | |
| Heat Treatment | Heat the sections for 5 min at 90-100°C in PB | | | | | | | | | | | | Heat the sections for 5 min at 90-100°C in citrate buffer | |
| SDS Treatment | Place sections in **1% SDS** solution for 5 min on the orbital shaker at RT | | | | No treatment | | | | | | | | | |
| Wash | Wash sections in PB 3 times, 5 min each, on the orbital shaker at RT | | | | No washes | | | | | | | | | |
| Blocking Solution Recipe | **3% milk** and 0.2% tween in PB | | 2% BSA and 0.2% tween in PB | | 2% BSA and 0.2% tween in PB | | | **3%** **milk** and 0.2% tween in PB | | | **5%** **milk** and 0.2% tween in PB | | **3% milk** and 0.2% tween in PB | 2% BSA and 0.2% tween in PB |
|  | Add **2% NDS** | No NDS | Add **2% NDS** | No NDS | Add **2% NDS** | No NDS | | | | Add **2% NDS** | No NDS | | | |
| Blocking Incubation | 1 hour incubation in original block on the orbital shaker at RT | | | | | | | | | | | | | |
| Primary Antibody Solution | Dilute chicken anti-Netrin-1 antibody at 1:500 concentration and rabbit anti-TH antibody at 1:1000 concentration in block | | | | | | | | | | | | | |
|  | Use original block | | | | | | | | | | **3%** **milk** and 0.2% tween in PB | Use original block | | |
| Primary Antibody Incubation | Incubate sections in primary antibody solution for 4 nights on the orbital shaker at 4°C | | | | | | | | | | | | | |
| Wash | Wash sections in PB 3 times, 5 min each, on the orbital shaker at RT | | | | | | | | | | | | | |
| Secondary Antibody Solution | Dilute goat anti-chicken (AF488) and donkey anti-rabbit (AF594) secondary antibodies at 1:500 concentrations in original block | | | | | | Use **HRP anti-chicken** | | Dilute goat anti-chicken (AF488) and donkey anti-rabbit (AF594) secondary antibodies at 1:500 concentrations in original block | | | | | |
| Secondary Antibody Incubation | Incubate sections in secondary antibody solution for 1 hour on the orbital shaker at RT | | | | | | | | | | | | | |
| H_2_O_2_ Solution Incubation | No H_2_O_2_ incubation | | | | | | Incubate sections in **H_2_O_2_ solution** for 10 min on the orbital shaker at RT. Then place the sections in water for **an instant** | | No H_2_O_2_ incubation | | | | | |
| Wash | Wash sections in PB 3 times, 5 min each, on the orbital shaker at RT | | | | | | | | | | | | | |
| Mount and Coverslip | Mount sections on gel-coated slides and cover-slip with DAPI | | | | | | | | | | | | | |

**Abbreviations:**

PBS: Phosphate buffered saline;

PB: Phosphate buffer;

SDS: Sodium dodecyl sulfate;

BSA: Bovine Serum Albumin;

NDS: Normal Donkey Serum;

RT: Room temperature;

TH: Tyrosine Hydroxylase;

AF: Alexa Fluor;

HRP: Horseradish Peroxidase;

DAPI: 4’, 6-diamidino-2-phenylindole


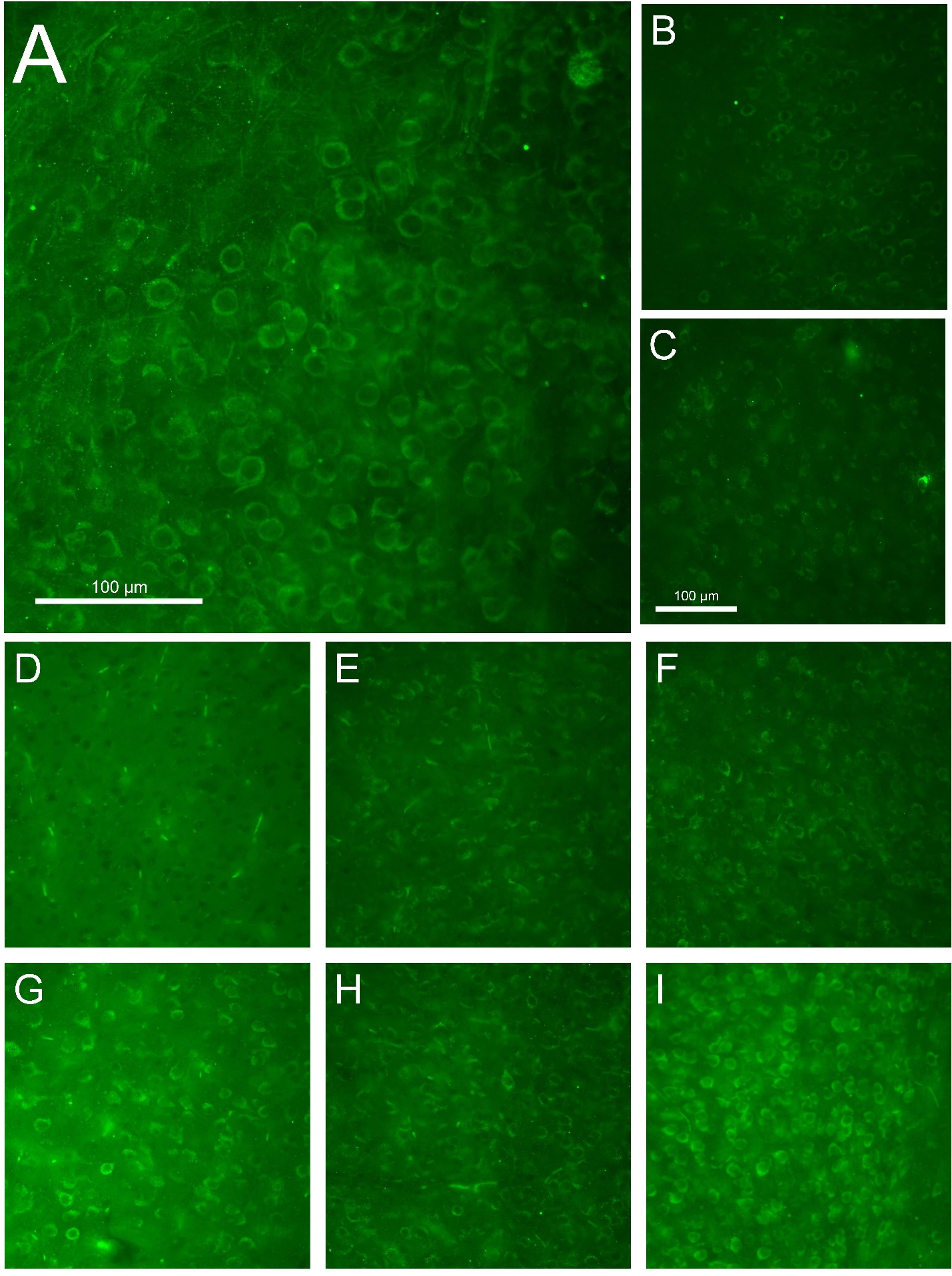


**Figure 1.** Netrin-1-labelled cell bodies in the lateral septum from Round 3 protocol variants. ***A***, Heat treatment in PB & SDS (Variant 4). ***B***, Heat treatment in PB (Variant 6). ***C***, Heat treatment in citrate buffer (Variant 14). ***D***, Heat treatment in PB & HRP-secondary antibody (Variant 7). ***E***, Heat treatment in PB & 3% milk block (Variant 9). ***F***, Heat treatment in PB, 5% milk block, & 3% milk primary antibody solution (Variant 11). ***G***, Heat treatment in PB & 3% milk / 2% NDS block (Variant 10). ***H***, Heat treatment in PB, SDS, & 3% milk block (Variant 2). ***I***, Heat treatment in PB, SDS, & 2% NDS block (Variant 3). ***A***, Variant 4 produces optimal SNR. ***B, C***, Heat treatment yields Netrin-1 IF signal with poor SNR. ***E-G***, Milk as blocking agent strengthens Netrin-1 IF signal but also has poor SNR. ***A, H, I***, SDS treatment optimizes SNR. Netrin-1 IF was viewed under green fluorescence at x 40 magnification.

Round 3 indicated that the SNR of Netrin-1 labelling was enhanced in all of the variants which included the 1% SDS treatment. Furthermore, the SNR was optimized when SDS was combined with the heat treatment (variant 4). Hence, we surmised that the SDS treatment is an effective antigen retrieval method.

The fourth round of testing protocol variations aimed to determine whether heat was necessary for an optimal Netrin-1 IF signal in the presence of SDS treatment. Citrate buffer and 2% NDS were also included in round 4 (Table 3, Figure 2).

**Table 3: Round 4 Protocol Variants**

| **Variants**  **Steps** | **1** | **2** | **3** | **4** | **5** |
| --- | --- | --- | --- | --- | --- |
| Collect | Collect brain sections in PBS | | | | |
| Wash | Wash sections in PB 3 times, 5 min each, on the orbital shaker at RT | | | | |
| Heat Treatment | **No Heat** | | Heat the sections for 5 min at 90-100°C in **PB** | Heat the sections for 5 min at 90-100°C in **citrate buffer** | |
| SDS Treatment | Place sections in 1% SDS solution for 5 min on the orbital shaker at RT | | | | |
| Wash | Wash sections in PB 3 times, 5 min each, on the orbital shaker at RT | | | | |
| Blocking Solution Recipe | 2% BSA and 0.2% tween in buffer solvent | 2% BSA, **2% NDS** and 0.2% tween in buffer solvent | 2% BSA and 0.2% tween in buffer solvent | | 2% BSA, **2% NDS** and 0.2% tween in buffer solvent |
| Blocking Incubation | 1 hour incubation in block on the orbital shaker at RT | | | | |
| Primary Antibody Solution | Dilute chicken anti-Netrin-1 antibody at 1:500 concentration and rabbit anti-TH antibody at 1:1000 concentration in block | | | | |
| Primary Antibody Incubation | Incubate sections in primary antibody solution for 4 nights on the orbital shaker at 4°C | | | | |
| Wash | Wash sections in PB 3 times, 5 min each, on the orbital shaker at RT | | | | |
| Secondary Antibody Solution | Dilute goat anti-chicken (AF488) and donkey anti-rabbit (AF594) secondary antibodies at 1:500 concentrations in block | | | | |
| Secondary Antibody Incubation | Incubate sections in secondary antibody solution for 1 hour on the orbital shaker at RT | | | | |
| Wash | Wash sections in PB 3 times, 5 min each, on the orbital shaker at RT | | | | |
| Mount and Coverslip | Mount sections on gel-coated slides and cover-slip with DAPI | | | | |

**Abbreviations:**

PBS: Phosphate buffered saline;

PB: Phosphate buffer;

SDS: Sodium dodecyl sulfate;

BSA: Bovine Serum Albumin;

NDS: Normal Donkey Serum;

RT: Room temperature;

TH: Tyrosine Hydroxylase;

AF: Alexa Fluor;

DAPI: 4’, 6-diamidino-2-phenylindole


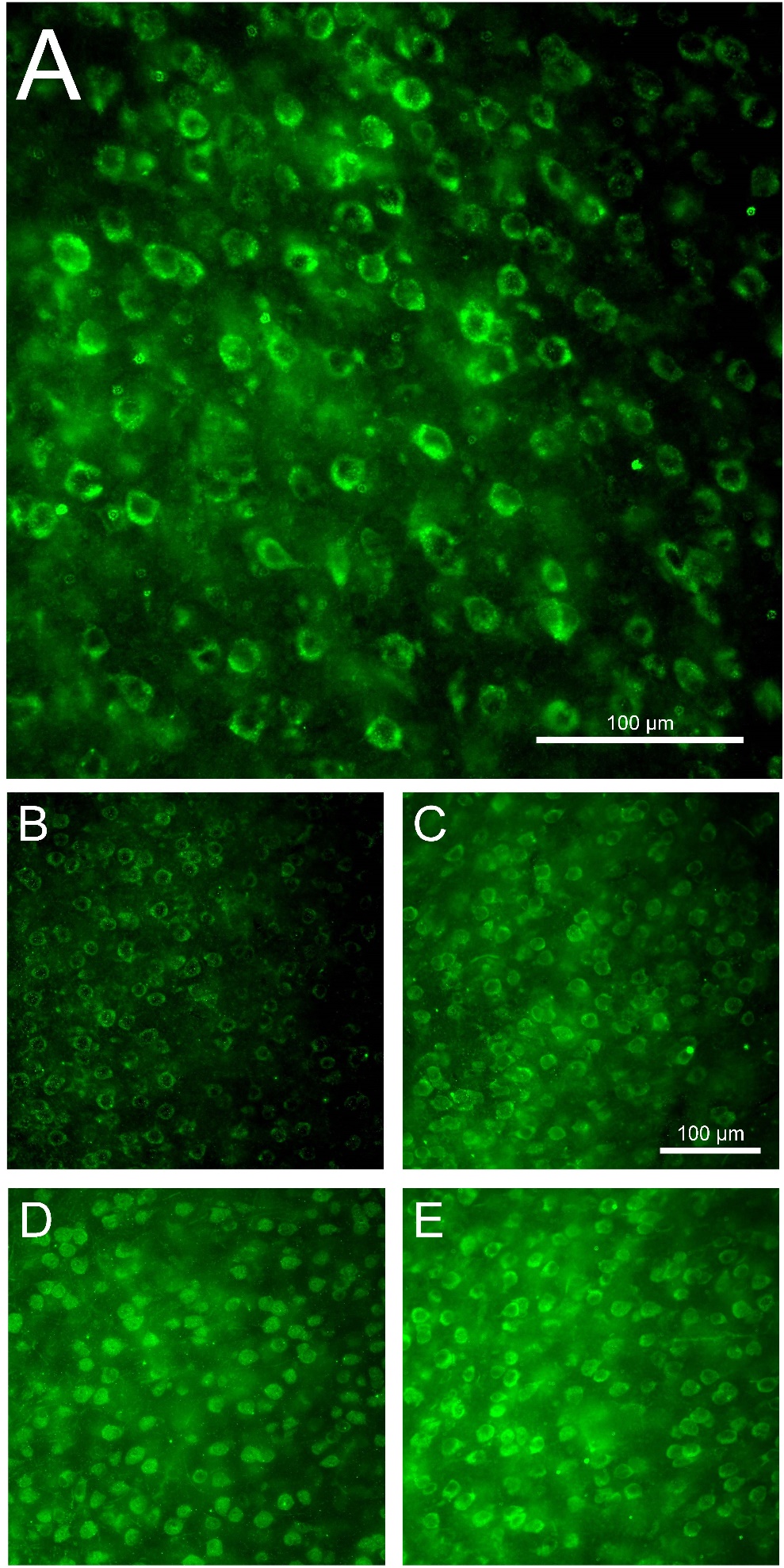
**Figure 2.** Netrin-1-labelled cell bodies in the lateral septum from Round 4 protocol variants. ***A***, SDS without heat (Variant 1). ***B***, SDS & 2% NDS block without heat (Variant 2). ***C***, SDS and heat treatment in PB (Variant 3). ***D***, SDS and heat treatment in citrate buffer (Variant 4). ***E***, SDS, 2% NDS block, & heat treatment in citrate buffer (Variant 5). ***A, B***, Heat treatment is not necessary for Netrin-1 IF signal, and Variant 1 produces optimal SNR. Netrin-1 IF was viewed under green fluorescence at x 40 magnification.

The fourth round of testing indicated that not only was the presence of heat not necessary for SDS treatment to produce Netrin-1 IF signal, but that the SNR of Netrin-1 labelling was improved when SDS was not combined with heat. NDS did not improve the SNR. A fifth round of testing was performed to determine if Netrin-1 signal in the presence of SDS treatment could be enhanced by an HRP-labeled secondary antibody or H_2_O_2_ incubation (Table 4).

**Table 4: Round 5 Protocol Variants**

| **Variant**  **Steps** | **1** | **2** | **3** |
| --- | --- | --- | --- |
| Collect | Collect brain sections in PBS | | |
| Wash | Wash sections in PB 3 times, 5 min each, on the orbital shaker at RT | | |
| SDS Treatment | Place sections in 1% SDS solution for 5 min on the orbital shaker at RT | | |
| Wash | Wash sections in PB 3 times, 5 min each, on the orbital shaker at RT | | |
| Blocking Solution Recipe | 2% BSA and 0.2% tween in PB | | |
| Blocking Incubation | 1 hour incubation in block on the orbital shaker at RT | | |
| Primary Antibody Solution | Dilute chicken anti-Netrin-1 antibody at 1:500 concentration and rabbit anti-TH antibody at 1:1000 concentration in block | | |
| Primary Antibody Incubation | Incubate sections in primary antibody solution for 4 nights on the orbital shaker at 4°C | | |
| Wash | Wash sections in PB 3 times, 5 min each, on the orbital shaker at RT | | |
| Secondary Antibody Solution | Dilute goat anti-chicken (AF488) and donkey anti-rabbit (AF594) secondary antibodies at 1:500 concentrations in block | | Use **HRP anti-chicken** |
| Secondary Antibody Incubation | Incubate sections in secondary antibody solution for 1 hour on the orbital shaker at RT | | |
| H_2_O_2_ Solution Incubation | No H_2_O_2_ incubation (this step was skipped) | Incubate sections in **H_2_O_2_ solution** for 10 min on the orbital shaker at RT. Then place the sections in water for **an instant** | |
| Wash | Wash sections in PB 3 times, 5 min each, on the orbital shaker at RT | | |
| Mount and Coverslip | Mount sections on gel-coated slides and cover-slip with DAPI | | |

**Abbreviations:**

PBS: Phosphate buffered saline;

PB: Phosphate buffer;

SDS: Sodium dodecyl sulfate;

BSA: Bovine Serum Albumin;

RT: Room temperature;

TH: Tyrosine Hydroxylase;

AF: Alexa Fluor;

HRP: Horseradish Peroxidase;

DAPI: 4’, 6-diamidino-2-phenylindole

Ultimately, HRP and H_2_O_2_ treatments did not enhance the efficacy of SDS. After five rounds of experimenting with different variations of antigen retrieval methods and blocking solution solvents, it was concluded that the final alterations to our group’s standard IHC protocol are as follows:

1. Use PB instead of PBS.
2. Prior to blocking incubation, place the sections in 1% SDS solution for 5 minutes on an orbital shaker at RT.
