# Supplementary material for: An optimized immunohistochemistry protocol for detecting the guidance cue Netrin-1 in neural tissue
Source: MethodsX. 2017 Dec 13;5:1–7. doi: 10.1016/j.mex.2017.12.001 (PMC5737949; doi:10.1016/j.mex.2017.12.001)
Supplement: Supplementary file 2 [file mmc2.docx]

**Supplementary Materials 2, Solutions used in this study**

- Heparinized Saline: 9 g sodium chloride and 1 mL heparin saline (1000 USP Units/mL) in 100 mL double distilled water (ddH_2_O).
- Monobasic stock (0.2 M): 13.9 g monobasic anhydrous (NaH_2_PO_4_) ***or*** 15.6 g monobasic dihydrous (NaH_2_PO_4_•2H_2_O) in 500 mL ddH_2_O.
- Dibasic stock (0.2 M): 28.4 g dibasic anhydrous (Na_2_HPO_4_) ***or*** 53.65 g dibasic heptahydrate (Na_2_HPO_4_•7H_2_O) in 500 mL ddH_2_O.
- Phosphate-buffered saline (PBS): PBS is a 10-fold dilution of 10x PBS (Premixed PBS Buffer, 10x, pH 7.0; Roche Diagnostics) in ddH_2_O.
- Citrate buffer (0.1 M): 0.0265% citric acid, 0.257% sodium citrate, and 0.05% tween in ddH_2_O; pH=6.0.
- Phosphate buffer (PB): 9.5% monobasic stock and 40.5% dibasic stock in ddH_2_O; pH~7.4; stored at RT.
- Tris buffer (TB): 12.1% tris base in ddH_2_O; pH~7.2-7.4; stored at RT.
- Tris-buffered saline (TBS): TBS is a 10-fold dilution in ddH_2_O of 10x TBS: 2.42% tris base and 0.03% 5M NaCl in ddH_2_O; pH~7.5; stored at RT.
- 4% Paraformaldehyde: 4 g paraformaldehyde per 100 mL PBS; pH~7.3; stored at 4°C.
- H_2_O_2_ solution: 24.8% H_2_O_2_ and TSA Tyramide Reagent at a concentration of 1:200 in PBS.
